# Analysis of Malnutrition among Children under Five Years across Contrasting Agroecosystems of Northwest Ethiopia: Application of Structural Equation Modeling

**DOI:** 10.3390/nu16081208

**Published:** 2024-04-18

**Authors:** Biruk Yazie Wubetie, Atsushi Tsunekawa, Nigussie Haregeweyn, Mitsuru Tsubo, Zerihun Nigussie, Taye Minichil Meshesha, Takeshi Abe

**Affiliations:** 1 The United Graduate School of Agricultural Sciences, Tottori University, 4-101 Koyama-Minami, Tottori 680-8553, Japan; 2 College of Agriculture and Environmental Sciences, Bahir Dar University, Bahir Dar P.O. Box 5501, Ethiopia; 3 Arid Land Research Center, Tottori University, 1390 Hamasaka, Tottori 680-0001, Japan; 4 International Platform for Dryland Research and Education, Tottori University, 1390 Hamasaka, Tottori 680-0001, Japan; 5 School of Civil and Water Resources Engineering, Debre Markos Institute of Technology, Debre Markos University, Debre Markos P.O. Box 269, Ethiopia

**Keywords:** child malnutrition, dietary intake, structural equation modeling, dryland, Ethiopia

## Abstract

Child malnutrition remains a public health challenge in developing countries, but a comprehensive understanding of its burden and its determinants in specific local contexts is generally lacking. This study examined the prevalence of malnutrition and its determinants among children aged <5 years across contrasting agroecosystems in northwest Ethiopia. A community-based cross-sectional study involving 400 respondents was employed. Data were collected through semi-structured questionnaires and anthropometric measurements, complemented with focus group discussions and key informant interviews. The direct and indirect effects of the determinants of malnutrition were examined using structural equation modeling (SEM). The overall prevalence of child malnutrition, measured using the Composite Index of Anthropometric Failure, was 49%, with notable variation across agroecosystems (from 36.1% [midland with red soil] to 59% [lowland and valley fragmented]). Disease experience had significant positive direct effects on malnutrition. Dietary intake had negative and significant total (direct and indirect) effects on malnutrition, partially mediated through disease experience. Serial mediation in SEM analysis revealed significant indirect relationships between malnutrition and food security, feeding and care practices, household environment, health services, maternal diet, maternal empowerment, household wealth, and nutrition-sensitive agricultural practices. In conclusion, child malnutrition was highly prevalent and higher among children in the lowland and valley fragmented agroecosystem, characterized by unfavorable agro-climatic conditions, lower wealth status, poor health services access, and higher disease (particularly malaria) exposure. This study demonstrates the dynamics and multifaceted nature of malnutrition, highlighting the importance of considering geographical differences when planning interventions for childhood malnutrition and its determinants.

## 1. Introduction

Malnutrition remains a significant global public health challenge despite concerted global efforts to enhance child nutrition. As of 2022, the statistics reveal a distressing reality: globally, over one-fifth of children aged <5 years were stunted (amounting to 148.1 million children), and 45 million children were affected by wasting [1]. Even more alarming is the fact that approximately 5.2 million children aged <5 years died due to malnutrition-related complications, with nearly half of these fatalities attributed to malnutrition [1]. The prevalence of malnourished children is disproportionately concentrated in Asia and Africa, with these regions bearing the brunt of the crisis. For instance, by 2020, more than half of all stunted and more than two-thirds of all wasted children aged <5 years lived in Asia, and two of every five stunted and more than one-quarter of all wasted children aged <5 years lived in Africa [2].

Echoing the global trend, child malnutrition remains a pressing public health issue in Ethiopia. The latest Ethiopian Demographic and Health Survey (EDHS) underscores this reality, revealing that 38% of children aged <5 years across Ethiopia are stunted, with an alarming 46% of children aged <5 years in the Amhara region being stunted. In addition, approximately 24% and 10% of these children are underweight and wasted, respectively [3]. The ramifications of child malnutrition are profound, accounting for approximately 28% of child mortality, 16% of primary school repetition, an 8% reduction in the workforce, and an annual economic cost of ETB 55.5 billion, equivalent to approximately 16.5% of the country’s gross domestic product [4].

Addressing the burden of child malnutrition requires a comprehensive understanding of its determinants within specific contexts, including at the individual, household, community, region, and environmental levels [5]. The framework conceptualized by the United Nations International Children’s Emergency Fund (UNICEF) identifies three types of determinants of malnutrition: basic, underlying, and immediate [6]. Empirical studies conducted globally confirm that a child’s dietary intake and disease experiences constitute immediate causes of malnutrition [7,8,9,10,11]. Similarly, studies conducted in various developing countries, including Ethiopia, highlight the significant association between child malnutrition and underlying determinants such as food security, feeding and care practices, health services, and household environments (water, sanitation, and hygiene) [7,8,10,11,12,13,14,15,16,17,18,19,20,21,22,23,24,25,26,27]. In addition, these studies also highlight the significant role of basic factors, such as maternal education, media exposure, training, nutrition-sensitive agricultural practices, agroecosystem characteristics, and household wealth in child malnutrition.

The Ethiopian government has demonstrated a strong commitment to addressing child malnutrition, as evidenced by the formulation of numerous multisectoral programs, policies, and initiatives since 2008. These include the National Nutrition Program (NNP-I and NNP-II), Nutrition-Sensitive Agriculture (NSA) policy, Seqota Declaration, National Nutrition Policy and Strategy, and the recently developed Food-Based Dietary Guideline [28,29]. However, despite these efforts, child malnutrition persists in Ethiopia, particularly in the Amhara region, where nearly half of all children aged <5 years are reported to be stunted [3]. This underscores the need for further research to provide us with a comprehensive understanding of the prevalence of child malnutrition and its determinants.

Although previous studies have provided valuable insights into child malnutrition, significant research gaps remain, particularly with regard to the effects of agriculture and agroecosystem characteristics on child malnutrition, which are often overlooked in the existing literature. In addition, methodological advances are warranted to better capture the complex and multifaceted nature of malnutrition determinants [30]. To this end, we used structural equation modeling (SEM) techniques in the present study, leveraging the UNICEF conceptual framework, to analyze the hierarchical relationships between malnutrition and its determinants. Unlike conventional regression analysis, SEM offers a more nuanced understanding of these relationships by allowing for the measurement of latent variables and their direct and indirect effects on malnutrition. Furthermore, this study used the Composite Index of Anthropometric Failure (CIAF) to comprehensively assess the prevalence of child malnutrition, thereby mitigating the risk of underestimation associated with conventional single anthropometric indicators [31]. 

The aims of the present study were to comprehensively examine the prevalence of malnutrition and to explore its determinants among children aged <5 years in northwest Ethiopia. The findings hold significant implications for stakeholders involved in nutrition-sensitive and nutrition-specific intervention programs, facilitating a nuanced understanding of spatial variations across agroecosystems. Therefore, targeting and working on <5 years children is a great window of opportunity to tackle the intergenerational impact of malnutrition.

### Conceptual Framework

**Figure 1:**
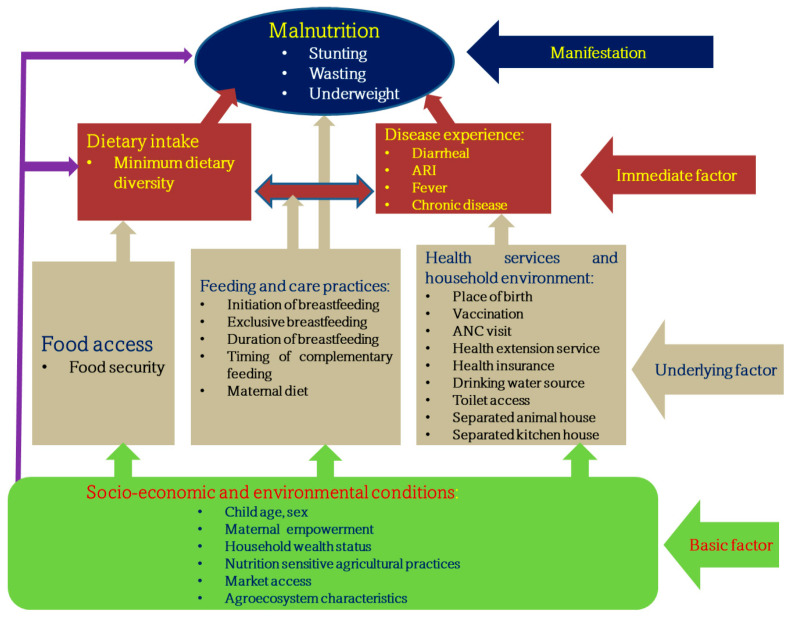
Modified conceptual framework on determinants associated with child malnutrition [6].

Drawing upon UNICEF’s conceptual framework and prior studies conducted in developing countries [6,32,33], this study adapts a conceptual framework tailored towards the Ethiopian context. The framework delineates the determinants and consequences of child malnutrition, operating across multiple levels of causation, from broad socioeconomic and environmental factors to immediate child-level factors. At its core, the framework distinguishes between basic, underlying, and immediate factors hypothesized to impact child malnutrition directly and indirectly, as shown in Figure 1. Immediate causes, such as exposure to disease and inadequate dietary intake, are identified as primary instigators of malnutrition in children. However, factors within each child’s household (e.g., insufficient access to food, poor maternal and child feeding practices, inadequate health services, and poor household environment encompassing issues like lack of safe water and sanitation) are recognized as proximal determinants significantly affecting the immediate causes of malnutrition. These household-level determinants represent proximal context indirectly affecting a child’s nutritional status. Beyond the household level, broader social factors encompassing socioeconomic status and environmental conditions are identified as distal causes, exerting their influence by shaping the quality and quantity of the resources available within households. Consequently, factors operating at the societal level are deemed as basic or root causes underlying child malnutrition.

## 2. Materials and Methods

### 2.1. Study Area

**Figure 2:**
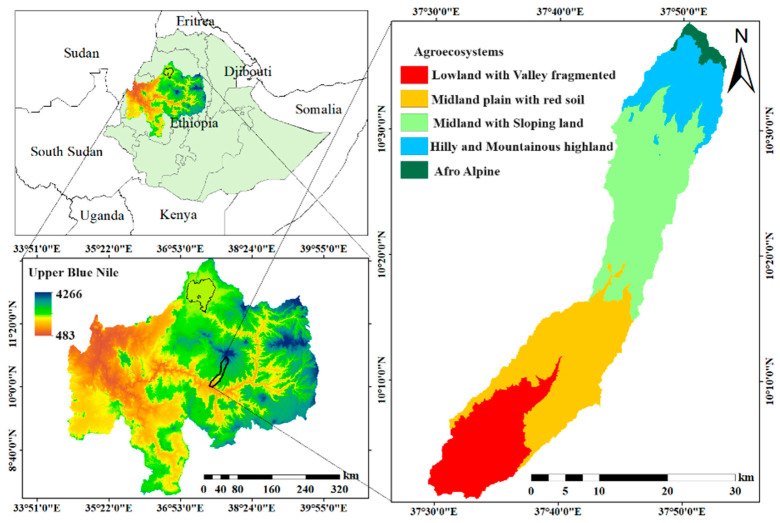
Maps showing the location of the study area.

The Chemoga watershed in the northwest part of Ethiopia is located within the Upper Blue Nile Basin (Figure 2), a region characterized by its diverse topography, ranging from highlands to lowlands. The Chemoga watershed is approximately 300 km northwest of Addis Ababa, the capital of Ethiopia, and covers a considerable land area with varying climatic and ecological conditions. The area is predominantly characterized by contrasting agroecosystems with diverse agricultural practices (Table 1), with altitudes ranging from 800 m above sea level (asl) in the lowlands and valley fragmented to higher peaks in the hilly and mountainous highlands (4200 m asl). The Chemoga watershed plays a vital role in the hydrology of the region, ultimately contributing to the flow of the Blue Nile River. Human activities, such as crop cultivation, livestock grazing, and settlement, are prevalent throughout the watershed, including along steep slopes, exerting pressure on the natural environment and contributing to land degradation [34]. Livelihoods in the area primarily rely on a mixed farming system integrating both crop cultivation and the rearing of livestock. Overall, the Chemoga watershed serves as an important ecological and hydrological component of the Upper Blue Nile Basin, playing a crucial role in sustaining local livelihoods and biodiversity.

### 2.2. Study Design and Sampling Procedures

The present study employed a cross-sectional study design and strategically used a multi-stage sampling method, with agroecosystem typology serving as a key stratification parameter. Initially, the Chemoga watershed was delineated with five distinct agroecosystems that covered three districts, namely Sinan, Gozamin, and Baso Liben. Subsequently, four representative kebeles (the smallest administrative units in Ethiopia, each classified according to their unique agroecosystem characteristics) were randomly chosen from four agroecosystems within three districts in the Chemoga watershed, namely Sinan (AEZ-3, AEZ-4), Gozamin (AEZ-1, AEZ-2), and Baso Liben (AEZ-1, AEZ-2). Since there are no human settlements within the afro-alpine agroecosystem (AEZ-5), it has been excluded from the sample selection process. In collaboration with local health extension workers, households with children aged <5 years were identified in the selected kebeles using the health post’s records as the sampling frame. Then, using a systematic random sampling approach, child–mother pairs were selected, with sample sizes allocated proportionately based on the number of children aged <5 years in each of the kebele. In cases where an individual mother had more than one child aged <5 years, one child was randomly selected for inclusion in the study.

The determination of the sample size adhered to the established formula for a single population proportion, ensuring appropriateness and rigor in the sampling process:

$$n=\frac{{\left(Z_{\alpha /2}\right)}^{2}\times p\left(q\right)}{d^{2}}$$

where *n* is the required sample size, *Z*_α/2_ is the upper point of α/2 (which has a value of 1.96 in the standard normal distribution with a 95% confidence level), p is the prevalence of the outcome of interest and was assumed to be 50% because there have been no previous studies in the area, q is calculated as (1−p), and d is the desired precision (5%). To achieve the desired precision level, a 5% non-response rate was considered. Finally, the study included 404 pairs of children aged <5 years along with their mothers.

### 2.3. Data Collection Methods

The study used a detailed household survey and anthropometric assessments involving 404 children aged <5 years with their mothers. The survey was conducted in June and July 2023. Data were collected through semi-structured questionnaires and anthropometric measurements. This was complemented with a focus group discussion and key informant interviews. The survey covered comprehensive information at various levels (child, maternal, household, and community), encompassing data on socioeconomic conditions, food security, dietary diversity, and relevant agricultural practices. The survey was administered by trained health and agricultural extension workers who were well acquainted with the sampled kebeles. The survey was closely supervised by the first author (BYW). To facilitate smooth administration, data collectors underwent a day-long training session. The survey was enriched with insights from key informant interviews, involving health and agricultural extension workers, alongside field observations.

### 2.4. Data Measurement

#### 2.4.1. Food Security

Food security status was assessed using the Household Food Insecurity Access Scale (HFIAS), a validated tool used in various developing countries [36]. This tool consists of nine standard generic questions addressing a household’s experiences over the past month across three domains of food insecurity: (i) anxiety and uncertainty regarding food availability, including worries about whether the household would have enough to eat; (ii) limitations in the variety and preferences of food accessed; and (iii) insufficient food intake in terms of meal sizes and their frequencies.

#### 2.4.2. Dietary Diversity 

Each child’s mother was asked about the foods that she and her child had eaten and the beverages they had consumed in the past 24 h. This study used the Food and Agriculture Organization of the United Nations (FAO) guideline, which measures dietary diversity across seven food groups for children and ten food groups for women (WDDS). Dietary diversity scores (DDSs) for children and women were calculated by summing the number of food groups consumed in the 24 h prior to the survey [37,38].

#### 2.4.3. Anthropometric Measurements

Anthropometric data were obtained by measuring children’s weight and height. Weight measurements in children were obtained without footwear and with minimal clothing, using the recommended UNICEF weighing scale to the nearest 0.1 kg. In cases where children refused to be weighed, their mothers would carry them and stand on the scale; the child’s weight was then determined by subtracting the mother’s weight from the combined weight of the mother and child. Scales were calibrated before weighing each child using a fixed 2 kg mass. For children who could stand independently, height was measured, without footwear, to the nearest 0.1 cm using a standard calibrated standing height board. Children were positioned against the standing board, ensuring that five body parts (heels, bat, buttocks, shoulders, and back of the head) touched the board. A measuring tape was fixed to the board, and the child stood with their head comfortably erect, aligning the lower border of the eye’s orbit with the external canal of the ear. Measurements were taken using a horizontally held thin wooden board by touching the top of the head. For children who could not stand independently, length measurements were taken to the nearest 0.1 cm by using a length board fixed with measuring tape and equipped with a headpiece and elongated foot piece. During the measurement, the child’s head touched the headpiece, and their heels were in contact with the foot piece. The hands remained relaxed throughout the measuring process. For each child, three consecutive measurements of weight and height/length were obtained, with the average of the three measurements used in the final analysis. The age of the child, in months, was determined from the date of birth (as reported by the mother) to the day of data collection.

### 2.5. Data Quality Assurance

Data quality was ensured through a meticulous design of the questionnaire and data collection process. Data collectors and supervisors underwent training on both data collection procedures and ethical considerations. A pre-test survey was conducted on 5% of households with similar backgrounds outside the targeted watershed to validate and tailor the questionnaire to local conditions. This iterative process validated the questionnaire and allowed for the customization of instruments. Supervisors checked on a daily basis to ensure completeness and consistency. In addition, the principal investigator (BYW) monitored the overall quality of the data collection as well as thorough entry, and proper management of all the data before the final analysis.

### 2.6. Data Management and Analysis 

Data were managed using SPSS version 28 (IBM Inc., Armonk, NY, USA). The data were then exported to Stata version 17 (Stata Corp, College Station, TX, USA) for further analysis by a combination of descriptive (percentage, mean, standard deviation [SD]) and inferential (chi-squared test, *t*-test, one-way analysis of variance [ANOVA]) statistics. The study primarily used SEM to measure the direct and indirect effects of risk factors on child malnutrition using SPSS AMOS version 29. In addition, a logistic regression model was used to analyze the direct influence of immediate factors only on child malnutrition. A household’s wealth index was computed through principal component analysis based on the main assets owned by the households (as shown in the Supplementary Materials). Standard deviation for the Z-score was computed for height-for-age, weight-for-age, and weight-for-height indices by comparison against the median of the World Health Organization (WHO) standards using the Emergency Nutrition Assessment (ENA) and WHO Anthro plus version 1.0.4 software.

### 2.7. Structural Equation Modeling

The hierarchical, complex, and interconnected nature of the determinants associated with child malnutrition requires a model that is capable of incorporating and estimating a set of equations simultaneously. This study used the SEM model, considering multiple levels (basic, underlying, immediate), with each level representing variables influencing one another that are treated as a dependent or response variable, as shown in the conceptual framework (Figure 1). SEM combines the statistical elements of multiple regression, confirmatory factor analysis, and path analysis [39]. This analysis was performed using AMOS version 29 software. It examines complex models with both measured and unmeasured variables (latent variables), allowing for the inclusion of endogenous, exogenous, and mediator variables. Unlike path analysis, SEM entertains latent variables alongside observed variables [39]. Mediation analysis was performed to predict the indirect effect of determinants on child malnutrition. In the context of the SEM framework, mediation is categorized as either full or partial [40]. Mediators can further be classified as single or multiple, single when only one variable lies in the causal pathway between exogenous and endogenous variables, and multiple when more than one mediator operates concurrently at the same stage in the structural model (termed “serial” or “sequential” mediation) [40]. Multigroup SEM analysis was conducted to estimate the effects on the structural model across different groups, offering insights into possible variations in relationships among variables across these groups.

SEM has two parts: a measurement model and a structure model. In the present study, model fitness for both models was determined using the goodness-of-fit index (GFI), comparative fit index (CFI), root mean square error of approximation (RMSEA), and standardized root mean squared residual (SRMR) [41]. Before running SEM, confirmatory factor analysis was performed to verify the model’s fit by excluding items with low factor loading. The measurement model was validated through reliability and validity assessments prior to the evaluation of causal relationships among variables in the structural model. The reliability test reflects the internal consistency of the observed variables through composite reliability, whereas validity examines the extent to which the indicator or a measure accurately reflects its constructs. Therefore, construct reliability and validity were determined using composite reliability (CR) and average variance extracted (AVE). The maximum likelihood estimation procedure was used in the model analysis. The estimation of the indirect effects used the product-of-coefficients test, whereas total effects were obtained by summing the direct and indirect effects, using bootstrap techniques to determine the level of significance. The Bayesian analysis method addressed missing data with the assumption of missing completely at random. Standardized and unstandardized coefficients, and their exponential (odds ratio), were used to estimate the direct, indirect, and total effects of predictors on child malnutrition. Ultimately, statistical significance was declared when *p*< 0.05. Generally, SEM provided a comprehensive analysis of the complex relationships influencing child malnutrition.

### 2.8. Operational Definitions and Key Variables of The Study

Malnutrition, as conceptualized in this study, encompasses various manifestations of undernutrition, namely stunting, wasting, and underweight. These conditions collectively contribute to a state of malnutrition, which is assessed through the CIAF, categorizing individuals into malnourished and not malnourished groups. 

The CIAF represents a comprehensive metric that evaluates malnutrition by combining multiple anthropometric failure indicators, including stunting, wasting, and underweight into a single index. It provides a more holistic measure of nutritional status than individual indicators alone. 

The main variables in this study are derived from the UNICEF conceptual framework, with some modification by customization to the Ethiopian context. Therefore, by reviewing the related literature, the key basic, underlying, and immediate factors are identified and included in the final model. For the sake of SEM analysis and interpretation in AMOS software, some multiple categorical measured variables, such as education, vaccination, wealth index, and agroecosystems, were changed to dummy categories (Table 2). 

### 2.9. Ethical Considerations

Before data collection, ethical approval was obtained from the Department of Rural Development and Agricultural Extension, College of Agriculture and Environmental Sciences, Bahir Dar University. During the survey, an official letter was sent to all districts to obtain permission for data collection. Verbal consent was obtained from all study participants before the commencement of the data collection. The data obtained from each study participant was kept confidential.

## 3. Results

### 3.1. Basic Sociodemographic Characteristics

Of the final 400 children aged <5 years included in the study (99% response rate), approximately 54% were boys and about 46% were girls (Table S1). The mean age of the children stood at 23 months, while the mean age of the mothers was 34 years. A significant majority (~95%) of mothers were married. Only 18% of the mothers had basic literacy skills; furthermore, only 10.3% had had the opportunity to participate in formal education (Table S1). Approximately one-quarter of the mothers received training pertaining to child nutrition, covering essential aspects such as infant and child feeding practices, and overall health awareness. Of note, 40% of the mothers were equal decision makers in their households, particularly on significant matters. Approximately two-thirds of the households fell within the bracket of poor to middle wealth, indicative of the prevalent economic challenges faced by the studied communities (Table S1). 

### 3.2. Food Security, Dietary Diversity, and Feeding Practices

As presented in Table 3, most children (61%) in this study were from food-insecure households. Most children (~90%) were exclusively breastfed up to 6 months of age, and approximately 92% were breastfed within 1 h of birth. However, nearly half the children (46%) were not introduced to complementary feeding within the recommended time of 6 months. For children, the mean(±SD) child DDS was 3.62 ± 1.03 (out of seven standard food groups), and only 43.5% achieved the minimum DDS (four or more food groups) in the 24 h prior to the survey. The number of children achieving minimum DDS differs significantly between the malnourished and normal nutritional status groups (Table 3). For the mothers, the mean minimum DDS was 4.45 ± 0.84 (out of 10 standard food groups), and approximately 40% met the minimum DDS (five or more food groups; Table 3).

**Figure 3:**
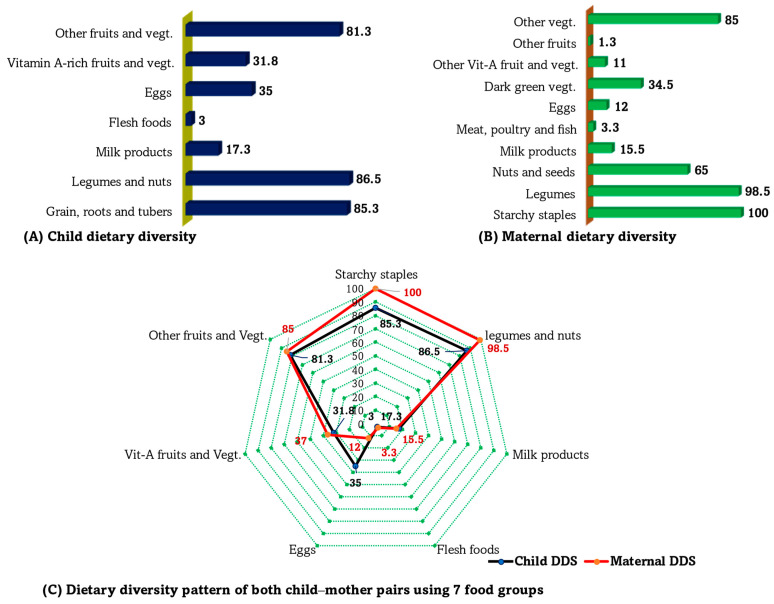
(**A**,**B**) Minimum dietary diversity among children (**A**) and mothers (**B**). (**C**) Consumption patterns among children and mothers based on 7 food groups.

As shown in Figure 3, starch staples (e.g., grains, roots, and tubers) formed a significant component of the daily diet, with approximately 85% of children and all mothers reporting the consumption of starch staples within the 24 h period preceding the survey. Legumes and nuts, as well as other fruits and vegetables (particularly onions), were the most frequently consumed food groups, whereas both children and mothers consumed notably less animal-based foods and vitamin A-rich fruits and vegetables (see Figure 3). There was a linear relationship between children and their respective mothers in the distribution of food group consumption (Figure 3C), although the primary focus of the comparisons was to determine intrahousehold variations in dietary intake due to possible misconceptions about child feeding habits in rural families.

### 3.3. Home Gardening as a Nutrition-Sensitive Agricultural Practice

The home gardening practices among the surveyed households were limited in scope. Approximately 10% of households cultivated micronutrient-rich fruits and vegetables, specifically dark green leafy and Vitamin A-rich varieties (Figure S1). Conversely, there was a relatively robust production of animal-based foods, although actual consumption remained constrained. The assessment of home gardening practices, based on the home gardening index derived through principal component analysis, revealed that approximately 30% of households had a higher level of engagement in home gardening activities aimed at enhancing both food security and household income (Figure S1).

### 3.4. Household Environment, Health Services, and Children’s Disease Experiences 

Regarding water, sanitation, and hygiene within households, slightly more than half (53.4%) of households had access to piped drinking water (Table 4). Furthermore, over three-quarters of households had their own toilets, separate kitchens, and animal housing that was detached from their primary living quarters. Approximately 69.5% of mothers adhered to the recommended minimum number of antenatal care (ANC) visits during pregnancy; however, approximately 27.5% of children included in the study were delivered at home without professional assistance. Nearly four of five children had received complete vaccinations. On average, the mothers of the surveyed children had had interactions with health extension workers twice in the previous year (Table 4). In addition, slightly over four-fifths of households had availed themselves of health insurance services. However, instances of fever and diarrheal diseases were reported for approximately one-fifth of children (Table 4).

### 3.5. Prevalence of Stunting, Wasting, and Underweight and Overall CIAF 

**Figure 4:**
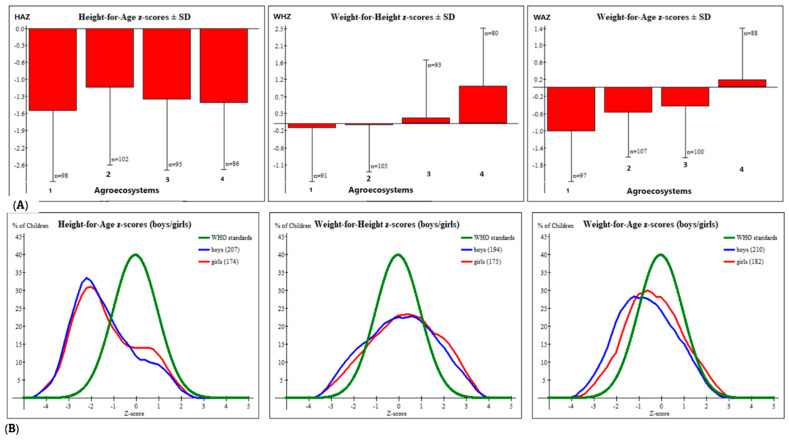
(**A**) Mean (±SD) height-for-age (HAZ), weight-for-height (WHZ), and weight-for-age (WAZ) Z-scores across agroecosystems. (**B**) Prevalence of stunting (HAZ), wasting (WHZ), and underweight (WAZ) in the study area compared with World Health Organization (WHO) standards.

The height-for-age, weight-for-age, and weight-for-height indices (stunting, underweight, and wasting, respectively) were compared with a WHO standardized normal distribution curve, which has a mean of zero and an SD of one. As indicated in Table 5, approximately 39% of children aged <5 years in this study have experienced stunting, with approximately 6% classified as severely stunted. The overall mean (± SD) of the height-for-age Z-score was –1.34 ± 0.35, with statistical analysis (ANOVA F = 2.43, *p* = 0.065) suggesting that there is no significant difference in mean scores for children across the four distinct agroecosystems (Figure 4A). The prevalence of wasting (weight for height) and underweight (height for age) was 9.5% and 11.5%, respectively, with approximately 1% of children experiencing severe forms of these conditions.

The ANOVA test revealed significant differences in the mean Z-scores for both weight-for-height (F = 21.36, *p* < 0.001) and height-for-age (F = 17.54, *p* < 0.001) indices across the agroecosystems.

**Figure 5:**
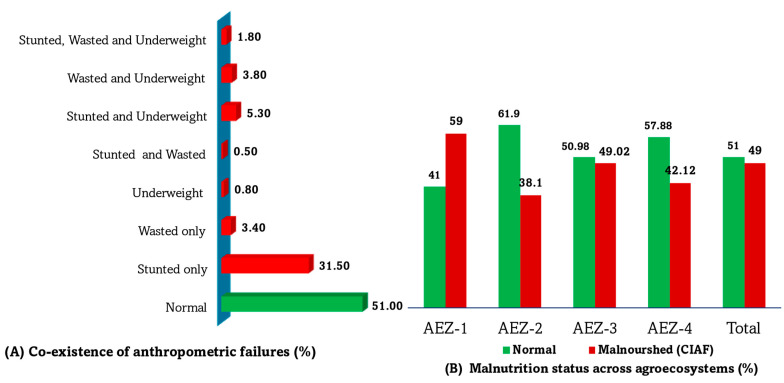
(**A**) Burden of the coexistence of indices of anthropometric failure among children. (**B**) Prevalence and distribution of overall malnutrition across agroecosystem zones (AEZ).

Based on the CIAF, nearly half the children in the study were malnourished. Of these, slightly less than one-third (31.5%) were stunted or had a short stature (Figure 5). Approximately 10% of children had double anthropometric failures (both stunting and either wasting or underweight simultaneously), whereas 2% had triple anthropometric failures (stunting, wasting, and underweight concurrently). Chi-squared analysis revealed statistically significant differences in the distribution of malnourished children across various AEZs (χ^2^ = 24.84; *p* < 0.001). Notably, the prevalence of malnutrition was higher in the lowland and valley fragmented agroecosystem (AEZ-1).

### 3.6. Determinants Associated with Child Malnutrition

**Figure 6:**
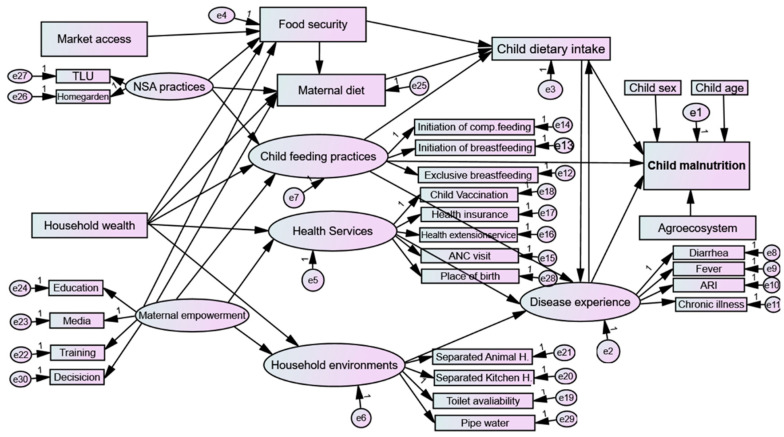
Conceptual structural equation modeling and its hypothesized path diagram. Circles in the SEM diagram indicate latent exogenous and endogenous variables, whereas rectangles represent observed or measured exogenous and endogenous variables. Arrows between circles and rectangles indicate the paths and relationship between variables, as denoted by their coefficients. 1 refers to fixed loading. ANC, antenatal care; ARI, acute respiratory infection; comp, complementary; NSA, nutrition-sensitive agriculture; TLU, livestock size by Tropical Livestock Unit; Decision, decision making; Media, media exposure.

SEM analysis was conducted at both the measurement and structural levels. The path diagram in Figure 6 illustrates detailed hypothetical relationships between exogenous and endogenous variables. 

#### 3.6.1. Measurement Model

Confirmatory factor analysis was used to test the measurement model. During this process, factor loadings for each item within every construct were examined, and items with low factor loadings were excluded. The fitness of the measurement model was assessed based on the CFI, with a threshold of ≥0.9, and the SRMR, with a threshold of <0.08. Construct reliability was evaluated using the CR, with a threshold of ≥0.7. The validity of the constructs was assessed through the AVE, with a threshold of ≥0.5. As indicated in Table 6, all the values met their respective acceptance thresholds, indicating that the constructs were reliable and valid, and the measurement model adequately fit the data. The anticipated outcome for the chi-square test of model fitness was non-significant. However, it was indeed significant as expected, due to its sensitivity to sample size during model testing. 

#### 3.6.2. Structural Model

The fitness of the structural model was evaluated using several indices (Table 7). The GFI yielded a value of 0.91, slightly surpassing the acceptable range of >0.9. The CFI returned a value of 0.78, falling below the desired threshold of >0.9. The RMSEA was calculated at 0.082, slightly exceeding the acceptable range of <0.08. However, the Chi-square divided by the degrees of freedom yielded a value of 3.71, which falls within the acceptable range of 3–5. Given these results, the model required modification to ensure that all the model fit statistics fell within their acceptable ranges. Therefore, model modifications were made iteratively until all indices met the criteria for a satisfactory fit. This process aimed to enhance the overall fitness of the structural model and improve its validity for the dataset under investigation. The model is very complex and involves more measured variables, which makes it difficult to be more fitted; however, the model is still good enough to predict the data. The main intention of SEM analysis is to verify a theoretical hypothesis rather than solely enhance a model’s goodness-of-fit. Pursuing improvements in fitness alone may result in inappropriate model configuration. Consequently, the model may fail to accurately represent reality, thus diminishing the persuasive power of the researcher’s argument.

#### 3.6.3. Direct Effects of Structural Model

**Figure 7:**
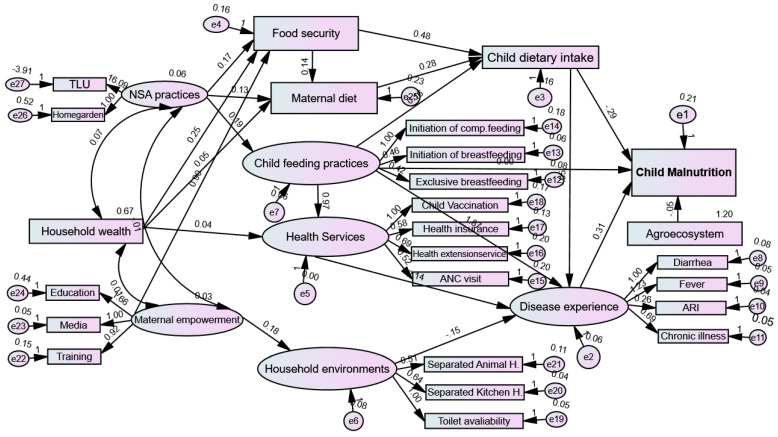
Direct effect estimates of modified SEM and its output paths diagram. Circles in the SEM diagram indicate latent exogenous and endogenous variables, whereas rectangles represent observed or measured exogenous and endogenous variables. Arrows between circles and rectangles indicate the paths and relationship between variables, as denoted by their coefficients. ANC, antenatal care; ARI, acute respiratory infection; comp, complementary; NSA, nutrition-sensitive agriculture; TLU, livestock size by Tropical Livestock Unit; Decision, decision making; Media, media exposure.

Figure 7 shows the finalized structural model following the re-specification of the conceptual model, incorporating adjustments based on modification indices to enhance the model’s fit. Table 8 provides insights into the direct effects within the model. Child dietary intake was found to have a significant negative direct effect on children’s malnutrition (β = −0.295, Exp(B) = 0.75, *p* = 0.001), indicating that an increase in dietary intake is associated with a decrease in the likelihood of malnutrition. Conversely, disease experiences as a risk factor had a significant positive effect on children’s malnutrition (β = 0.306, Exp(B) = 1.36, *p* = 0.009), suggesting that experiencing disease increases the likelihood of malnutrition. 

Furthermore, the model confirmed that agroecosystems had a significant negative effect on children’s malnutrition status (β = −0.051, Exp(B) = 0.60, *p* = 0.015), indicating that children in the lowland and valley fragmented agroecosystem (AEZ-1) were at higher risk of malnutrition than children in other agroecosystems. To keep the length of this paper to a minimum, the path estimates towards the other endogenous variables shown in Table 8 are not discussed further. However, the full direct effect results of the SEM analysis with regression path coefficients are provided (Table 8; Figure 7). To see the effect of agroecosystem variation on child malnutrition, binary logistic regression was conducted by controlling or adjusting for other immediate factors that are shown in the conceptual framework. Using AEZ-1 as the reference group, the adjusted odds ratios for the likelihood of being malnourished were 0.424 (*p* = 0.027) in AEZ-2, 0.609 (*p* = 0.207) in AEZ-3, and 0.274 (*p* = 0.001) in AEZ-4 (Table S2).

#### 3.6.4. Indirect and Total Effects in the Mediation Analysis of the Structural Model

Serial mediation analysis was applied because the mediators have a kind of hierarchal or sequential nature (Figure 7). Therefore, the serial mediation analysis, detailed in Table 9, elucidated several significant pathways, contributing to the relationship between various factors and child malnutrition. Maternal empowerment had a negative and significant direct effect on malnutrition (β = −0.06, Exp(B) = 0.94, *p* = 0.012). This effect was fully mediated via food security, maternal diet, household environment, minimum child dietary diversity, and child disease experiences. Similarly, the effects of NSA practices (β = −0.03, Exp(B) = 0.97, *p* = 0.038) and household wealth status (β = −0.12, Exp(B) = 0.89, *p* = 0.001) on malnutrition were fully mediated and significantly negative, indicating no direct effect. Food security, maternal diet, household environment, and health service utilization were identified as underlying causes of malnutrition, with only indirect and significantly negative effects on child malnutrition (Table 9). Child feeding and care practices were considered as both underlying and immediate causes of malnutrition, with no significant direct effect (β = −0.001, Exp(B) = 0.99, *p* = 0.995), but a negative and significant indirect effect (β = −0.059, Exp(B) = 0.941, *p* = 0.045) mediated by dietary intake and child disease experiences. Child dietary intake (minimum dietary diversity) had a partially mediated effect on malnutrition via disease experiences, with a significant total effect. In addition, the SEM output confirmed that agroecosystem and disease had a fully unmediated significant relationship with malnutrition. 

Detailed standardized coefficients for indirect and total effects resulting from mediation analysis within the entire structural model are provided in Tables S3 and S4, respectively.

#### 3.6.5. Agroecosystem-Based Multigroup SEM Analysis

**Figure 8:**
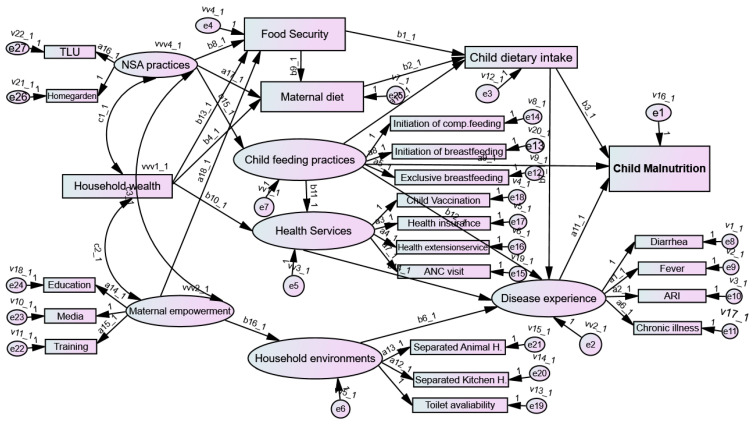
Path diagram for multigroup SEM analysis between agroecosystem zone (AEZ)-1 and other AEZs. Circles in the SEM diagram indicate latent exogenous and endogenous variables, whereas rectangles represent observed or measured exogenous and endogenous variables. Arrows between circles and rectangles indicate the paths and relationship between variables, as denoted by their coefficients. ANC, antenatal care; ARI, acute respiratory infection; comp, complementary; NSA, nutrition-sensitive agriculture; TLU, livestock size by Tropical Livestock Unit; Decision, decision making; Media, media exposure.

In this study, a multigroup SEM analysis was conducted to compare two distinct groups, resulting in the formation of four binary groups: AEZ-1 vs. other AEZs (AEZ-2, -3, -4); AEZ-2 vs. other AEZs; AEZ-3 vs. other AEZs; and AEZ-4 vs. other AEZs. Figure 8 presents the structural model, wherein each parameter is uniquely named across the groups, with ‘a’ and ‘b’ followed by group numbers representing the relationships between the variables. The comparison analysis, facilitated by the chi-squared difference test (*x^2^* = 108.53, *p* < 0.001), revealed a significant difference between AEZ-1 and other AEZs when contrasting the structural weights constrained model with the unconstrained model-free model. However, although this test indicated a significant difference in the group overall, it did not pinpoint where specific paths in the model differed. 

Further examination revealed that specific paths, such as b1_1, b9_1, b10_1, b14_1, and a11_1, exhibited significant differences between AEZ-1 and other AEZs (Figure 8; Table 10). Overall, the comparison between the overall structural weight model and the unconstrained model demonstrated significant differences, implying variations in the effects of paths between AEZ-1 and other AEZs. However, the chi-squared difference test did not reveal significant differences in the structural model weights between AEZ-2 and other AEZs (χ^2^ = −420.38), AEZ-3 and other AEZs (χ^2^ = −403.492), and AEZ-4 and other AEZs (χ^2^ = −671.56). 

In the multigroup SEM analysis, each structural relationship was individually constrained to determine if specific relationships differed across the groups. The results of constraining the relationship ‘a11_1’ revealed a significant chi-squared difference (χ^2^ = 6.412, *p* < 0.01), indicating that the effects of disease on malnutrition vary significantly between children living in AEZ-1 and those living in other AEZs. Furthermore, the model results showed significant chi-square difference tests in the specific relationship paths of the constrained “b1_1 (χ^2^ = 12.589, *p* < 0.01), b9_1 (χ^2^ = 16.69, *p* < 0.01), b10_1 (χ^2^ = 3.32 *), and b14_1 (χ^2^ = 48.04, *p* < 0.01)” between children living in AEZ-1 and those living in other AEZs. However, there were no significant differences between these two groups in the remaining paths in the structural model, despite the overall structural model showing significant differences between the two groups (Figure 8; Table 10).

## 4. Discussion

In this study, we investigated the prevalence of child malnutrition. Among children aged <5 years in this study, approximately 39% were stunted (i.e., short stature for their age). This figure aligns closely with the national prevalence of stunting reported in the latest EDHS of 38%. However, it is of note that the prevalence of stunting in this study was slightly lower than that reported specifically for the Amhara region of 46% [3]. The difference between our findings and those for the Amhara region could be attributed to the wider geographical coverage of the latter, encompassing significant spatial variations in the environment and agricultural productivity. Such discrepancies in environmental conditions and agricultural productivity may give rise to diverse socioeconomic conditions, which, in turn, could influence the prevalence of child malnutrition. The prevalence of wasting and underweight in this study was lower than the national averages reported in the EDHS of 10% and 24%, respectively [3], as well as lower than those reported for the Tigray region of northern Ethiopia [42]. These findings suggest that stunting, representing a chronic form of malnutrition, is the predominant anthropometric failure affecting children in the Chemoga watershed.

In addition, we found a high prevalence of CIAF in this study, with approximately half the children experiencing some form of anthropometric failure. The prevalence of CIAF surpassed that of single conventional indices of anthropometric failure, such as stunting, wasting, and underweight. Significant variations in the prevalence of CIAF were observed across the four AEZs in the study. The highest prevalence of CIAF was reported in the lowland and valley fragmented agroecosystem (AEZ-1), whereas the lowest was recorded among children from AEZ-2 (midland with red soil). The higher prevalence of child malnutrition in AEZ-1 is consistent with findings reported previously [9,24,43,44,45], and it may be due to unfavorable agroecosystem conditions, resulting in lower agricultural productivity and weak socio-economic status. Of note, a lower proportion of children from the hilly and mountainous highland agroecosystem (AEZ-4) were malnourished. This could be attributed to their dietary practices, characterized by the high consumption of potatoes, which are high in calories, as a staple food. Moreover, the environmental advantages of cold temperatures in AEZ-4 may contribute to the maintenance of better health conditions among children, as suggested by previous studies [20,46]. However, this stands in stark contrast to the lower prevalence reported in AEZ-4 in previous studies [24,44], underscoring the complex interplay among environmental factors, dietary practices, and nutritional outcomes across different AEZs. A possible reason for the apparent discrepancy could be differences in how children’s nutritional status was assessed: unlike the previous studies, which used a conventional single index of anthropometric failure, we used CIAF in the present study. This finding provides significant insights into specific geographic variations in child malnutrition, offering advantages in identifying the neediest subpopulations and allocating scare resources for further nutrition-sensitive and nutrition-specific interventions not only in the study area, but also in other similar environments [8].

In this study, we used SEM to investigate causal relationship between various determinants (including basic, underlying, and immediate factors) and child malnutrition. Our analysis revealed negative and statistically significant direct and indirect effects of child dietary intake on malnutrition. Specifically, the odds ratio for the total effect (combining direct and indirect effects) of minimum dietary diversity on malnutrition was 0.75. This implies that with each unit increase in a child’s DDS, the odds of experiencing malnutrition decrease by 0.75 units. In simpler terms, achieving minimum dietary diversity is associated with a notable 25% reduction in the relative risk of malnutrition. This underscores the significance of dietary diversity in mitigating the likelihood of malnutrition among children. Furthermore, our analysis revealed that the effect of dietary diversity on malnutrition was partially mediated through the exposure of children to diseases. This suggests that adequate diversification in a child’s diet, encompassing foods from at least four of the seven food groups, as recommended by the FAO [37], could enhance nutrient adequacy, subsequently lowering children’s susceptibility to diseases and thereby reducing the risk of malnutrition. However, it is concerning that more than half of the children in this study failed to meet the minimum DDS, particularly lacking in the consumption of animal-based foods and fruits and vegetables. This finding echoes the findings of prior studies conducted not only in Ethiopia [8,24,47] but also across Sub-Saharan Africa [12,32] and in countries like South Africa [48] and Ghana [49], highlighting the challenge of improving nutritional status without ensuring adequate dietary diversity among children in the region.

Our analysis also revealed a direct and significant positive effect of disease experiences on malnutrition. Specifically, the unmediated effect of disease exposure on malnutrition was quantified with an odds ratio, indicating that for each unit increase in a child’s exposure to disease, the odds of being malnourished increase by 1.36 units. Consequently, children experiencing disease are more likely to suffer from malnutrition compared with their healthier counterparts. This finding aligns with prior research, including reports from the EDHS [3] and UNICEF [50], and studies conducted in northwest Ethiopia [13]. It underscores the detrimental impact of various diseases on children’s nutritional status, which can be attributed to factors such as loss of appetite, the malabsorption of nutrients, and overall malfunctioning of metabolic processes.

In parallel with this descriptive result regarding child malnourishment, SEM and binary logistic regression analysis were conducted to measure the influence of agroecosystem characteristics on child malnutrition. The result of these analyses confirmed that agroecosystem is significantly associated with malnutrition based on logistic regression output, compared with a child from the lowland and valley fragmented agroecosystem (AEZ-1), where the risk of being malnourished was reduced by 42% and 27% for children from the midland with red soil (AEZ-2) and hilly and mountainous highland (AEZ-4) agroecosystems, respectively (Supplementary Table S1). Studies from the Amhara region in Ethiopia [24] and from Indonesia [11] and Ecuador [51] provide evidence of the effects of agroecosystem and agroecology on an individual’s nutritional status.

Based on logistic regression output, the risk of being malnourished was reduced by 42% and 27% for a child from midland with plain lands and from hilly and mountainous highland agroecosystems, respectively, compared with a child from the lowland and valley fragmented agroecosystem zone (Supplementary Table S1). Evidence from Amhara region, Ethiopia [24], Indonesia [11], and Ecuador [51] also indicated the influence of agroecosystem and agroecology on individual’s nutritional status. The higher risk of malnutrition in AEZ-1 could be related to higher climate change vulnerability and the resulting lower agricultural productivity and socioeconomic status compared with the midland with red soil agroecosystem (AEZ-2) [35]. 

Similarly, when comparing children in AEZ-1 to those in AEZ-4, the observed variations in nutritional status could be due to differences in dietary habits. In AEZ-4, potatoes are a staple calorie-rich food consumed daily, potentially leading to additional weight gain and an increase in anthropometric indices. Another factor contributing to differences between AEZ-1 and AEZ-4 could be the environmental conditions. AEZ-4 is characterized by cold temperatures, which could provide an advantage for maintaining health compared with AEZ-1; in AEZ-1, children are exposed to higher temperatures and may be more susceptible to heat stress, which aggravates the burden of child malnutrition. This is supported by findings of studies conducted in Ethiopia [20] and Uganda [52]. Moreover, in multigroup SEM analysis used to investigate differences in path coefficients across AEZs in which agroecosystem was used as a moderator variable after being recategorized into four dichotomous groups, the Chi-squared test for the whole structural model across all groups was only significant for the first group (AEZ-1 vs. others [AEZ-2, -3, and -4]). Further analyses were performed for AEZ-1 vs. others to identify differences in specific path coefficients, in addition to the whole structural model. This analysis highlighted specific entry points for further nutrition-sensitive and -specific interventions to reduce the burden of childhood malnutrition.

In this study, childcare and feeding practices, treated as a latent variable, were hypothesized to have direct and indirect effects on child malnutrition. However, SEM analysis indicated insignificant direct effects of child feeding practices on malnutrition, thus not supporting our prior hypothesis. In contrast, previous studies conducted in Ethiopia [7,8,13,53], Nigeria [54], and India [55] demonstrated an effect of child feeding practices, particularly breastfeeding and complementary feeding, on child nutritional status using various regression analyses other than SEM. Despite the lack of a direct effect, the indirect effects of care and feeding practices on malnutrition in the present study were statistically significant and negative. These indirect effects were mediated through a child’s dietary intake and disease. Children who initiated breast feeding within 1 h of birth were exclusively breastfed up to 6 months of age and who were introduced to semisolid and solid complementary food in a timely manner were less likely to have a history of illness and inadequate dietary diversity. Consequently, this led to a reduced risk of childhood malnutrition.

Household food security is a key underlying determinant of malnutrition [56,57]. Mediation analysis in the present study revealed that household food security had a significant negative indirect effect on child malnutrition. Children from food-secure households were less likely to be malnourished than children from food-insecure households. When a child is from a food-secure household, the relative risk of being malnourished is decreased by 15% compared with a child from a food-insecure household. This finding is supported by the findings of a study in Indonesia that used SEM analysis [11]. 

The household environment, particularly factors related to water, sanitation, and hygiene, plays a pivotal role in influencing child malnutrition. As per our prior hypothesis, the result of the SEM analysis confirmed a negative and statistically significant indirect effect of household environment on malnutrition through the mediator of disease experience. In this study, a child from a healthy household environment (i.e., with their own toilet, separated kitchen facilities, and housing animals separately) was less likely to be malnourished compared with children from less ideal household environments. This finding aligns with that of studies conducted in Ethiopia [58] and Indonesia [11] using SEM analysis, as well as other studies conducted in Ethiopia [7,59], Nigeria [54], and India [55]. A potential explanation for the role of the household environment in child malnutrition is that an unhealthy household environment could facilitate the rapid spread of infectious diseases. This result emphasizes the need to address not only direct determinants of nutrition but also broader household environmental determinants, through which their impact is indirectly manifested [60].

In the SEM analysis, treating health services as a ‘latent variable’ provides a comprehensive understanding of their influence on child malnutrition. Considering child vaccination status, ANC visits, household health insurance, and health extension services as components to measure the latent construct ‘health services’ emerge as a vital strategy in the broader effort to combat child malnutrition. In the present study, the effect of health services on child malnutrition was negative and statistically significant and was fully mediated through disease experience. This aligns with previous studies conducted in Ethiopia [59,61] and Indonesia [11] and suggests that ensuring adequate health services utilization can play a crucial role in improving child health and consequently reducing the prevalence of malnutrition. 

The findings of this study underscore the crucial influence of maternal empowerment and maternal diet in the complex framework of child malnutrition. In addition to working on a child directly, it is important to ensure the empowerment of mothers alongside addressing their dietary needs. The path coefficient in the SEM analysis revealed that the indirect effect of maternal diet on child malnutrition was negative and statistically significant. A child’s mother who achieved the minimum of five of the ten food groups in a population can be used as a proxy indicator for better micronutrient adequacy [62]. Other studies have also consistently shown an association between inadequate maternal dietary diversity and an increased likelihood of child malnutrition [58,60,62,63]. This may be due to the influence of a mother’s cultural preferences, adherence to food taboos, limitations regarding food access, and economic constraints, ultimately affecting a child’s dietary diversity and their nutritional outcome. This dual approach of targeting both children and their mothers are pivotal in devising a comprehensive strategy to improve child nutrition.

Similarly, SEM analysis revealed that maternal empowerment significantly influenced malnutrition at as far distance as the basic factors. When a mother’s empowerment was increased by one unit, the likelihood of her child being malnourished was reduced by 0.94 units, or their risk of malnutrition decreased by 6%. This may be attributed to empowered mothers being more likely to have high decision-making power in the household regarding child food and health care services. This finding aligns with the results of community-based cross-sectional studies conducted in Ethiopia [24,33] and Tanzania [16], as well as the findings of a systematic review study [64]; collectively, the results of these studies showed that maternal empowerment serves as a distant basic determinant of child nutrition, encompassing aspects such as education level, media exposure, and decision making power regarding a child’s health and nutrition. 

Household wealth was found to have significant negative indirect effects on malnutrition. According to serial mediation analysis, when the wealth index increased by 1 unit, the odds of malnutrition among children decreased by 0.88 units. In this study, children from poor households were more likely to experience any forms of malnutrition than children from rich households. The effect of household wealth was fully mediated, and the hypothesis in the conceptual structural model is supported. This finding is supported by other studies [11,12,22,32,33,54,65,66,67].

Most importantly, this study indicated that the probability of a child being malnourished was significantly lower among children from households who practiced better NSA, which was measured in this study as home gardening practices and livestock production. The effects of NSA practices on child malnutrition were indirect and fully mediated via underlying and immediate factors. These findings are consistent with some previous studies [19,68,69]. The likely explanation for the finding is that NSA practices play a crucial role in addressing the problem of food insecurity (the physical and economic accessibility of food) at a household level, which is one of the key underlying determinants of child malnutrition. In subsistence rural families, NSA practices, particularly home gardening, allow families to cultivate a variety of fruits and vegetables, as well as animal-based foods, which promotes a diverse and nutritious diet for children. This diversity ensures that children receive the essential micronutrients necessary for their growth and development [28].

## 5. Conclusions

In the present study, the prevalence of child malnutrition across four agroecosystems in northwest Ethiopia was unacceptably high. The prevalence of child malnutrition varied spatially and was particularly high among children residing in the lowland and valley fragmented agroecosystem compared with other areas. Furthermore, a high proportion of both children and their mothers did not meet the required minimum dietary diversity, in particular, the consumption of animal-based foods and fruits and vegetables was limited. SEM analysis was used as a robust analytical tool to address the hierarchical nature of risk factors of child malnutrition. The SEM analysis confirmed the direct effect of immediate factors and uncovered the indirect effects of basic and underlying determinants for child malnutrition by adapting the modified version of UNICEF’s conceptual framework for malnutrition. Based on the results of the SEM analysis, disease experience and minimum dietary diversity have significant direct effects on child malnutrition, whereas the direct effects of childcare and feeding practices were not statistically significant. Underlying factors such as health service utilization, household environment, feeding practices, household food security, and minimum maternal dietary diversity, as well as basic factors such as household wealth, maternal empowerment, and NSA practices, had significant indirect effects on child malnutrition. The holistic analytical approach used in this study allowed for a more comprehensive understanding of the dynamics and multifaceted nature of malnutrition, providing valuable insights into the implementation of sustainable and targeted nutrition-sensitive and specific-interventions. Based on our findings, we also recommend considering geographical differences in the burden of childhood malnutrition when planning interventions.

## Figures and Tables

**Table 1:** General characteristics of the agroecosystems in Chemoga watershed.

Agroecosystem ^A^	Mean Annual Temperature (°C)	Mean Annual Rainfall (mm)	Altitude (m asl)	Farming System
AEZ-1	15.7–27.4	996.29	800–1400	Sorghum–maize–teff
AEZ-2	10.4–22.7	1251.10	1400–2400	Wheat–maize–teff
AEZ-3	10–22	1282.40	2400–2800	Wheat–barley
AEZ-4	8.8–21.2	1399.30	2800–3800	Potato–barley
AEZ-5	5.4–18.1	1434.5	3800–4200	Choke protected area

Rainfall and temperature data averaged over 1983–2018 from the Ethiopian Metrological Agency. ^A^ Locations of the different agroecosystem zones (AEZs) are shown in Figure 2. AEZ-1, lowland with valley fragmented; AEZ-2, midland plain with red soils; AEZ-3, midland with sloping land; AEZ-4, hilly and mountainous highland; AEZ-5, afro alpine. Sources: own survey and [35].

**Table 2:** Description and measurement of predictor variables.

Variable	Indicators	Measurement	Level
Latent variable			
Women empowerment	Education level	1, can read and write; 0, otherwise	Basic factors
	Nutrition related training taken	1, yes; 0, otherwise	
	Media exposure	1, yes; 0, otherwise	
NSA practices	Home gardening	1, good practice; 0, poor practice	
	Livestock in TLU	Number of livestock in TLU	
Feeding and care practices	Initiation of BF	1, started within 1 h; 0, otherwise	Underlying factors
	Exclusive BF	1, yes; 0, otherwise	
	Initiation of CF	1, started on time; 0, otherwise	
Household environment	Toilet availability	1, yes; 0, otherwise	
	Kitchen house	1, separated from living quarters; 0, otherwise	
	Animal house	1, separated from living quarters; 0, otherwise	
Health services	Vaccination	1, fully vaccinated; 0, otherwise	
	ANC visit	1, ≥4 visit; 0, otherwise	
	Health insurance	1, they have; 0, otherwise	
	Extension service	Number of contacts per year	
Disease experience	Diarrheal disease	1, experienced it; 0, otherwise	Immediate factor
	Fever	1, experienced it; 0, otherwise	
	ARI	1, experienced it; 0, otherwise	
	Chronic illness	1, experienced it; 0, otherwise	
Observed variables			
Wealth status	Wealth index	1, rich; 0, poor	Basic
Food access	Food security	1, food secure; 0, food insecure	Underlying
Maternal diet	Minimum WDDS	1, ≥5 food groups; 0, otherwise	Underlying
Child dietary intake	Minimum CDDS	1, ≥4 food groups; 0, otherwise	Immediate
Agroecosystem	AEZ	1, AEZ-2,3,4; 0, AEZ-1	Basic
Malnutrition	CIAF	1, malnourished; 0, otherwise	Outcome

AEZ, agroecosystem zone; ANC, antenatal care; ARI, acute respiratory infection; BF, breastfeeding; CDDS, dietary diversity score for children; CF, complementary feeding; CIAF, Composite Index of Anthropometric Failure; NSA, nutrition-sensitive agricultural practices; TLU, Tropical Livestock Unit; WDDS, dietary diversity score for women.

**Table 3:** Household food security, maternal and child diet, and their feeding practices.

Variables	Malnutrition (%)			*x* ^2^
	Yes	No	Total	
Household food security status				
Food insecure	38.3	22.5	60.8	48.30 **
Food secure	10.8	28.5	39.3	
Exclusive breastfeeding				
No	6.0	4.5	10.5	1.25
Yes	43.0	46.5	89.5	
Initiation of breastfeeding				
Not within 1 h of birth	6.0	2.0	8.0	9.41 **
Within 1 h of birth	43.0	49.0	92.0	
Initiation of complementary feeding				
Not timely	24.0	21.5	45.5	1.88
Timely started	25.0	29.5	54.5	
Minimum child DDS				
Not achieved	37.4	19.1	56.5	55.25 **
Achieved	11.5	32.0	43.5	
Minimum maternal DDS				
Not achieved	28.2	31.8	60.0	3.68 *
Achieved	22.8	17.3	40.0	

* *p* < 0.05, ** *p* < 0.01; DDS, dietary diversity score.

**Table 4:** Household environment and health service utilization and disease experiences.

Variables	Indicators	Responses	Malnutrition (%)			*x* ^2^
			Yes	No	Total	
Household environment	Water source	Not pipe	21.1	25.6	46.6	2.19
		Pipe water	28.1	25.3	53.4	
	Toilet access	Open field	10.3	6.0	16.3	6.15 *
		Own toilet	38.8	45.0	83.8	
	kitchen house	Not separate	5.0	2.5	7.5	4.05 *
		Separate	44.0	48.5	92.5	
	Animal housing	Not separate	8.0	7.8	15.8	0.10
		Separate from living quarters	41.0	43.3	84.3	
Health services	ANC follow-up	<4 times	16.8	13.8	30.5	2.46
		≥4 times	32.3	37.3	69.5	
	Place of birth	Home	15.8	11.8	27.5	4.16 *
		Health institution	33.3	39.3	72.5	
	Child vaccination	Not at all	1.3	1.3	2.5	4.09 *
		Partially vaccinated	10.8	7.2	18.0	
		Fully vaccinated	37.0	42.5	79.5	
	Health insurance	No	10.8	8.3	19.0	2.16
		Yes	38.3	42.8	81.0	
	No. of health extension contact in past 12 months ^A^		2.02 ± 2.4	2.9 ± 2.5	t = −3.69 **	
Disease experiences	Diarrhea	No	36.5	46.3	82.8	18.4 **
		Yes	12.5	4.8	17.3	
	ARI	No	46.0	49.5	95.5	2.35
		Yes	3.0	1.5	4.5	
	Fever	No	37.8	44.8	82.5	7.93 **
		Yes	11.3	6.3	17.5	
	Chronic disease	No	41.8	49.0	90.8	14.08 **
		Yes	7.2	2.0	9.3	

^A^ Data are given as the mean ± SD. * *p* < 0.05, ** *p* < 0.01. ANC, antenatal care; ARI, acute respiratory infection.

**Table 5:** Prevalence (%) of malnutrition based on Z-scores.

Forms of Malnutrition	Severity of Malnutrition		All Children (Z-Score < −2)	Mean ± SD Z-Score
	Moderate (Z-Score −2 to −3)	Severe (Z-Score < −3)		
Stunting (HAZ)	32.8 (22.0–43.6)	6.3 (3.4–9.2)	39.1 (30.3–47.9)	−1.34 ± 0.35
Wasting (WHZ)	8.4 (4.6–14.4)	1.1(0.4–2.1)	9.5 (4.6–14.4)	0.21 ± 0.49
Underweight (WAZ)	10.2 (5.2–15.2)	1.3 (0.3–2.2)	11.5 (5.7–17.2)	−0.51 ± 0.23
CIAF			49	

Unless indicated otherwise, data show the percentage of children in each group, with 95% confidence intervals given in parentheses. CIAF, Composite Index of Anthropometric Failure; HAZ, height-for-age Z-score; WAZ, weight-for-age Z-score; WHZ, weight-for-height Z-score.

**Table 6:** Reliability and validity tests of constructs and model fit statistics for the measurement model.

Constructs/Latent Variables	No. Items	CR	AVE	CFI	SRMR	*x^2^* (*p* Value)
NSA practices	2	0.71	0.58	0.99	0.01	58.55 (0.01)
Maternal empowerment	3	0.78	0.56	0.99	0.023	76.77 (0.001)
Feeding and care practice	3	0.70	0.45	0.99	0.01	60.14 (0.001)
Household environment	3	0.77	0.53	0.98	0.001	190.33 (0.001)
Health services	4	0.76	0.50	0.99	0.01	49.4 (0.001)
Diseases experience	4	0.80	0.51	0.94	0.05	21.25 (0.001)

AVE, average variance extracted; CFI, comparative fit index; CR, composite reliability; NSA, nutrition-sensitive agriculture; SRMR, standardized root mean squared residual.

**Table 7:** Model fitness indices for the modified structural model.

Indices	*x* ^2^	*df*	*x*^2^/*df*	RMSEA	CFI	GFI
Structural model	867.90	234	3.71	0.082	0.75	0.91
Acceptable range	-	-	3–5	<0.08	>0.9	>0.9

CFI, comparative fit index; GFI, goodness-of-fit index; RMSEA, root mean square error of approximation.

**Table 8:** Standardized and unstandardized estimates for direct effects on structural model.

Direct Effect Paths			S (β)	β	S.E.	Exp(B)	*p*
Child malnutrition	**←**	Child dietary intake	–0.295	–0.295	0.048	0.75	0.001
	**←**	Feeding and care practice	–0.001	–0.059	0.130	0.55	0.647
	**←**	Agroecosystem zone	–0.113	–0.051	0.021	0.60	0.015
	**←**	Disease experience	0.151	0.306	0.118	1.36	0.009
Child dietary intake	**←**	Food security	0.475	0.478	0.041	1.61	0.001
	**←**	Maternal diet	0.278	0.282	0.041	1.33	0.001
	**←**	Feeding and care practice	0.082	0.158	0.100	1.17	0.115
Disease experience	**←**	Household environment	–0.182	–0.155	0.056	0.86	0.006
	**←**	Child dietary intake	–0.104	–0.051	0.028	0.95	0.068
	**←**	Feeding and care practice	1.958	1.865	1.137	6.46	0.101
	**←**	Health services	–2.172	–2.14	1.51	0.12	0.063
Food security	**←**	NSA practices	0.084	0.172	0.086	1.19	0.046
	**←**	Wealth status	0.422	0.254	0.029	1.29	0.001
	**←**	Maternal empowerment	0.262	0.903	0.216	2.47	0.001
Maternal diet	**←**	Wealth status	0.089	0.053	0.035	1.05	0.125
	**←**	Food security	0.145	0.144	0.055	1.15	0.009
	**←**	NSA practices	0.064	0.13	0.09	1.14	0.148
Feeding and care practice	**←**	NSA	0.174	0.186	0.066	1.20	0.005
Household environment	**←**	Maternal empowerment	0.144	0.177	0.124	1.19	0.155
Health services	**←**	Wealth status	0.138	0.042	0.021	1.04	0.049
	**←**	Feeding and care practice	0.99	0.970	0.168	2.64	0.001

NSA, nutrition-sensitive agriculture policy; S (β), standardized coefficient; Exp(B), exponential of coefficient, **←**, refers to the direction of effect paths.

**Table 9:** Mediation effects (standardized) on child malnutrition in the structural model.

Exogenous Variables	Direct Effect		Indirect Effect		Total Effect		Mediation Analysis
	β	Exp(B)	Β	Exp(B)	β	Exp(B)	
NSA practices	–	–	−0.03	0.97 *	−0.03	0.97 *	Full mediation
Maternal Empowerment	–	–	−0.06	0.94 *	−0.06	0.94 *	Full mediation
Wealth status	–	–	−0.12	0.88 **	−0.12	0.88 *	Full mediation
Agroecosystem	−0.11	0.89 *	–	–	−0.11	0.89 *	Not applicable
Food security	–	–	−0.16	0.85 **	−0.16	0.85 **	Full mediation
Maternal diet	–	–	−0.09	0.91 **	−0.09	0.91 **	Full mediation
Health services	–	–	−0.33	0.72 **	−0.33	0.72 **	Full mediation
Household environment	–	–	−0.03	0.97 *	−0.03	0.97 *	Full mediation
Child feeding and care	−0.001	0.99	−0.059	0.94 *	−0.06	0.94	Full mediation
Child dietary diversity	−0.30	0.74 **	−0.02	0.98 *	−0.32	0.73 **	Partial mediation
Disease experiences	0.15	1.16 **	–	–	0.15	1.16 **	Not applicable

* *p* < 0.05, ** *p* < 0.01; NSA, nutrition-sensitive agriculture.

**Table 10:** Agroecosystem as moderator in the multigroup SEM analysis.

Specific Relationship Paths		Group				
		AEZ-1		Other AEZs		Model Comparison *(x*^2^-Difference Test)
		β	Exp(B)	β	Exp(B)	
Malnutrition	**←**Child dietary intake	−0.14	0.87	−0.31	0.73 **	−19.47
	**←**Feeding and care practice	−0.03	0.97	−0.70	0.50	−19.83
	**←**Disease experience	3.04	20.91 **	0.295	1.34 **	6.412 **
Child dietary intake	**←**Food security	0.22	1.25 **	0.57	1.77 **	12.59 **
	**←**Maternal diet	0.41	1.51 **	0.21	1.23 **	2.93
	**←**Feeding and care practice	0.01	1.01	0.66	1.93	−19.96
Disease experience	**←**Household environment	0.04	1.04	0.001	1.01	0.34
	**←**Child dietary intake	−0.79	0.45	−0.03	0.76	−19.75
	**←**Feeding and care practice	0.01	1.01	−1.81	0.16	−16.41
	**←**Health services	−1.46	0.23 *	−0.83	0.44	48.04 **
Food security	**←**Wealth status	0.27	1.30 **	0.30	1.35 **	0.384
	**←**Maternal empowerment	0.03	1.03	0.91	2.48 **	1.814
Maternal diet	**←**Wealth status	0.140	1.15 **	0.07	1.08	1.17
	**←**Food Security	0.23	1.26 **	0.21	1.23 **	16.69 **
	**←**NSA	−0.15	0.86	0.001	1.001	1.50
Feeding and care practice	**←**NSA	−0.14	0.87	−0.002	0.99	0.80
Household environment	**←**Maternal empowerment	−0.01	0.99	0.29	1.34 **	1.53
Health services	**←**Wealth status	0.15	1.16 *	−0.03	0.97	3.32 *
	**←**Feeding and care practice	0.007	1.01	−0.05	0.95	−12.81
Overall model comparison (structural weight model vs. unconstrained model)						108.14 **

* *p* < 0.05, ** *p* < 0.01; AEZ, agroecosystem zone; NSA, nutrition-sensitive agriculture.

## Data Availability

The data presented in this study are available on request to the corresponding author.

## Supplementary Materials

The following supporting information can be downloaded at: https://www.mdpi.com/article/10.3390/nu16081208/s1, Table S1: Socio-demographic and economic characteristics of the respondents; Table S2: Binary logistic regression analysis output of direct effect of immediate factors on malnutrition; Table S3: Standardized indirect effect of structural model; Table S4: Standardized total effect of structural model; Figure S1: Home gardening practices as nutrition-sensitive agricultural practices in the households by percentage.
