# Supplementary material for: Analysis of Malnutrition among Children under Five Years across Contrasting Agroecosystems of Northwest Ethiopia: Application of Structural Equation Modeling
Source: Nutrients. 2024 Apr 18;16(8):1208. doi: 10.3390/nu16081208 (PMC11054005; doi:10.3390/nu16081208)
Supplement: Supplementary file 1 [file nutrients-16-01208-s001.zip › nutrients-2939811-supplementary.pdf]

Supplementary Materials

Table S1: Socio-demographic and economic characteristics of the respondents.

Characteristics		Agroecosystem Zones (%)				
		AEZ-1	AEZ-2	AEZ-3	AEZ-4	Total
Child sex	Girls	45.0	46.3	52.0	41.1	46.3
	Boys	55.0	53.7	48.0	58.9	53.8
Child age	0 – 6 months	18.0	10.2	16.7	3.3	12.3
	6 - 23 months	40.0	48.1	33.3	46.7	42.0
	24 - 59 months	42.0	41.7	50.0	50.0	45.8
Maternal education	No education	82.0	90.7	79.4	73.3	81.8
	Informal education	13.0	0.0	1.0	20.0	8.0
	Primary education	4.0	9.3	14.7	1.1	7.5
	Secondary & above	1.0	0.0	4.9	5.6	2.8
Marital status	Single	0.0	7.4	3.9	1.1	3.3
	Married	97.0	91.7	91.2	98.9	94.5
	Widowed	3.0	0.9	4.9	0.0	2.3
Household wealth	Poor	53.0	11.1	44.6	63.3	33.3
	Meddle	28.0	25.9	51.5	32.2	34.3
	Rich	19.0	63	4.0	4.0	32.3
Maternal Training	No	83.0	81.5	60.8	77.8	75.8
	Yes	17.0	18.5	39.2	22.2	24.3
Media exposure	No	97.0	88.9	92.2	82.2	90.3
	Yes	3.0	11.1	7.8	9.8	17.8
Decision making	No equal power	35.0	69.4	65	73.3	60.5
	Equal power	65.0	30.6	35	26.7	39.5
Maternal age (mean ±Sd)		31.53 (5.74)	34.37 (4.84)	32.17 (5.68)	34.52 (4.55)	33.13 (5.38)
Family size (mean ±Sd)		4.540	5.194	4.912	5.300	4.98

	(1.40)	(1.38)	(1.78)	(1.89)	(1.64)
Landholding size (mean ±Sd)	1.47<br>(1.04)	1.47<br>(.92)	0.45<br>(.54)	1.0<br>(1.17)	1.11<br>(1.03)
Livestock size (mean ±Sd)	3.18<br>(1.84)	7.25<br>(3.21)	2.70<br>(2.0)	2.36<br>(2.16)	3.97<br>(3.12)
Market distance (mean ±Sd)	93.08<br>(21.80)	85.0<br>(17.61)	34.80<br>(13.52)	42.0<br>(29.60)	64.54<br>(33.2)

Table S2: Binary logistic regression analysis output of direct effect of immediate factors on malnutrition.

	B	S.E.	Exp(B)	Sig.
Agroecosystem				0.004
Agroecosystem(1)	-0.858	0.389	0.424	0.027
Agroecosystem(2)	-0.495	0.393	0.609	0.207
Agroecosystem(3)	-1.295	0.377	0.274	<0.001
Exclusive breastfeeding(1)	0.158	0.292	1.171	0.588
Child dietary diversity (1)	-1.661	0.260	0.190	<0.001
Diarrheal disease(1)	0.583	0.366	1.791	0.111
Child fever(1)	-0.291	0.367	0.748	0.428
ARI(1)	0.302	0.598	1.352	0.614
Constant	0.877	0.682	2.403	0.198

Table S3: Standardized indirect effect of structural model.

Exogenous Variables	Endogenous Variables (Estimates with P-Value)							
	FA	MD	FC	HS	HE	CD	Disease	Malnutrition
NSA practices	-	0.012 (0.087)	-	0.18 (0.003)	-	0.08 (0.012)	-0.05 (0.007)	-0.03 (0.038)
Empowerment	-	0.049 (0.026)	-		-	0.17 (0.013)	-0.33 (0.066)	-0.06 (0.012)
Household wealth	-	0.06 (0.012)	-		-	0.24 (0.001)	-0.32 (0.002)	-0.12 (0.001)
Agroecosystem	-	-	-	-	-	-	-	-
Food access	-	-	-	-	-	0.04 (0.016)	-0.05 (0.111)	-0.16 (0.001)
Maternal diet	-	-	-	-	-	-	-0.03 (0.108)	-0.09 (0.001)
Feeding & care	-	-	-	-	-	-	-2.20 (0.001)	-0.059 (0.045)
Health services	-	-	-	-	-	-	-	-0.33 (0.009)
Household environment	-	-	-	-	-	-	-	-0.03 (0.024)
Child dietary intake	-	-	-	-	-	-	-	-0.02 (0.031)
Disease experience	-	-	-	-	-	-	-	-

Abbreviations on endogenous variables: FA-food access, MD-maternal diet, FC-feeding and care practice, HS-health service, HE-household environment, CD-child dietary intake, Disease-disease experience.

Table S4: Standardized total effect of structural model.

Exogenous variables	Endogenous variables (Estimates with P-value)							
	FA	MD	FC	HS	HE	CD	Disease	Malnutrition
NSA practices	0.08 (0.120)	0.08 (0.181)	0.17 (0.003)	0.18 (0.003)	-	0.08 (0.012)	-0.05 (0.007)	**-0.03 (0.038)**
Maternal Empowerment	0.34 (0.014)	0.05 (0.026)	-	-	0.12 (0.219)	0.17 (0.013)	-0.04 (0.066)	**-0.06 (0.012)**
Household wealth	0.42 (0.001)	0.15 (0.013)	-	0.14 (0.008)	-	0.24 (0.001)	-0.33 (0.001)	**-0.12 (0.001)**
Agroecosystem	-	-	-	-	-	-	-	**-0.11 (0.015)**
Food access	-	0.15 (0.022)	-	-	-	0.51 (0.001)	-0.05 (0.111)	**-0.16 (0.001)**
Maternal diet	-	-	-	-	-	0.28 (0.001)	-0.03 (0.108)	**-0.09 (0.001)**
Feeding & care	-	-	-	0.99 (0.001)	-	0.08 (0.233)	-0.23 (0.030)	**-0.06 (0.491)**
Health services	-	-	-	-	-	-	-0.99 (0.001)	**-0.33 (0.009)**
Household environment	-	-	-	-	-	-	-0.18 (0.043)	**-0.03 (0.024)**
Child dietary intake	-	-	-	-	-	-	-0.10 (0.113)	**-0.31 (0.001)**
Disease experience	-	-	-	-	-	-	-	**0.145 (0.009)**

Abbreviations of endogenous variables: FA-food access, MD-maternal diet, FC-feeding and care practice, HS-health service, HE-household environment, CD-child dietary intake, Disease-disease experience.

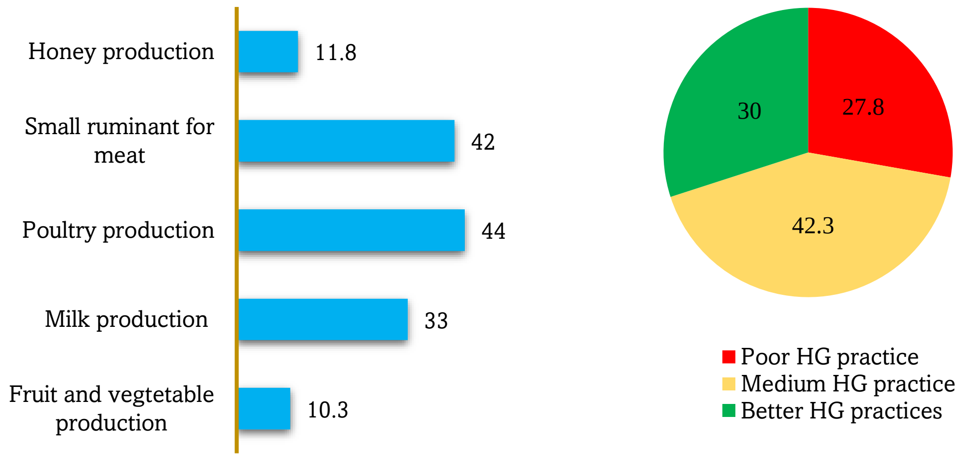

(A) Home-gardening practices (B) Home-gardening practices status

Figure S1: Home-gardening practices as nutrition sensitive agricultural practices in the households (%).
